# Obstetric management and pregnancy outcomes in pregnant women with kyphoscoliosis: a 15-year single-center retrospective cohort study

**DOI:** 10.1186/s12884-026-09155-5

**Published:** 2026-04-25

**Authors:** Ilgaz Rafi Kadıoğlu, Hüseyin Ekici, Ali Han Bolat, Fırat Ökmen, Davut Güven, İdris Koçak

**Affiliations:** 1 Department of Obstetrics and Gynecology, Ondokuz Mayıs University School of Medicine, Samsun, Turkey; 2 Department of Obstetrics and Gynecology, Andırın State Hospital, Kahramanmaras, Turkey; 3 Department of Obstetrics and Gynecology, Ege University School of Medicine, Izmir, Turkey

**Keywords:** Kyphoscoliosis, Complicated pregnancy, Obstetric outcomes, Anesthetic management, Neonatal morbidity

## Abstract

**Background:**

Kyphoscoliosis is a complex spinal deformity that may pose significant obstetric, anesthetic, and neonatal challenges during pregnancy. The aim of this study was to systematically evaluate obstetric management strategies and pregnancy outcomes in women with kyphoscoliosis based on a 15-year clinical experience.

**Methods:**

This retrospective study included adult pregnant women with clinically and radiologically confirmed kyphoscoliosis who delivered at the Department of Obstetrics and Gynecology, Ondokuz Mayıs University Faculty of Medicine, a tertiary referral center, between 2010 and 2025. Maternal characteristics, deformity-related parameters (including Cobb angle and prior spinal surgery), obstetric outcomes, anesthetic management, and neonatal outcomes were analyzed. Deformity severity was categorized based on Cobb angle as mild, moderate, or severe. Given the limited sample size, analyses were primarily descriptive, with comparative statistics applied where appropriate.

**Results:**

A total of 33 pregnancies were included. All cases were associated with adolescent idiopathic scoliosis, with a mean Cobb angle of 24.9 ± 12.2° and 42% of patients had a history of spinal surgery prior to pregnancy. The mean gestational age at delivery was 36 weeks. Cesarean section was performed in 94% of cases, and general anesthesia was predominantly required. Obstetric complications included fetal growth restriction, hypertensive disorders, gestational diabetes, and amniotic fluid abnormalities. Maternal complications were rare; with ileus in two cases and intensive care unit admission required in three patients. Neonatal intensive care admission was observed, and three neonatal deaths occurred, primarily related to underlying neonatal conditions. Subgroup analyses suggested higher maternal complication and intensive care admission rates among patients with severe deformity.

**Conclusions:**

Kyphoscoliosis in pregnancy may be associated with increased obstetric and neonatal risks, even in cases with moderate deformity. Management in tertiary care centers with multidisciplinary planning appears essential.

## Background

 Kyphoscoliosis is defined as a complex spinal deformity characterized by the coexistence of lateral curvature in the coronal plane (scoliosis) and kyphotic curvature in the sagittal plane, resulting in three-dimensional spinal distortion [1]. Its prevalence in the general population ranges between 0.3% and 0.5%, with a female predominance approximately two to three times higher than in males [2]. As a consequence of the deformity, structural asymmetry of the thoracic cage develops, leading to reduced thoracic volume, restricted pulmonary expansion, and impaired cardiopulmonary function. Although kyphoscoliosis most commonly involves the thoracolumbar region, cervicothoracic spinal segments may also be affected.

The etiology of kyphoscoliosis is highly heterogeneous and may be congenital, idiopathic, neuromuscular, infectious, traumatic, or degenerative in origin [1]. The Cobb angle, measured on posteroanterior radiographs, is the standard parameter used to assess the severity of scoliosis [3]. The severity of the deformity is typically assessed using the Cobb angle, which holds prognostic value for both diagnosis and longitudinal follow-up [4]. 

Outside of pregnancy, the diagnostic workup for kyphoscoliosis typically involves a thorough clinical examination, pulmonary function testing, and comprehensive radiological imaging, predominantly full-spine standing radiographs to measure the Cobb angle. Management strategies depend on curve progression and range from conservative approaches, such as physical therapy and bracing for mild curves, to surgical interventions involving spinal fusion and instrumentation for severe deformities. The long-term implications of this pathology include chronic back pain, accelerated spinal degeneration, and in severe cases, progressive cardiopulmonary compromise, all of which necessitate lifelong multidisciplinary monitoring.

Severity based on the Cobb angle is commonly categorized as mild (10–20°), moderate (20–40°), and severe (> 40°). In line with this stratification, mild cases are generally managed with observation and physiotherapy, moderate cases with orthotic bracing, and severe cases may warrant surgical evaluation and intervention [4]. 

Pregnancy imposes substantial physiological stress on the cardiorespiratory system, resulting in diaphragmatic elevation, decreased functional residual capacity, and increased oxygen consumption. In pregnant women with kyphoscoliosis and restrictive thoracic deformity, these changes may lead to reduced respiratory reserve and, in severe cases, maternal hypoxemia and increased perinatal risk [5]. Furthermore, kyphoscoliosis may alter pelvic skeletal anatomy, potentially impeding fetal descent and vaginal delivery; spinal deformity may also cause technical difficulties in the administration of regional anesthesia and contribute to increased cesarean delivery rates [6, 7]. Nevertheless, data regarding obstetric and neonatal outcomes in pregnancies complicated by kyphoscoliosis remain limited, with existing evidence largely derived from small, single-center retrospective series.

In this study, we aimed to analyze maternal and neonatal outcomes in pregnant women diagnosed with kyphoscoliosis, to identify pregnancy- and delivery-related complications in this population, and to compare our findings with the existing literature in order to develop clinically relevant recommendations for obstetric management.

## Materials and methods

This retrospective study was conducted at the Department of Obstetrics and Gynecology, Ondokuz Mayıs University Faculty of Medicine. Pregnant women aged ≥ 18 years who delivered at our tertiary referral center between January 2010 and December 2025 and had a clinically and radiologically confirmed diagnosis of kyphoscoliosis were included in the study. The study protocol was reviewed and approved by the Ondokuz Mayıs University Clinical Research Ethics Committee (OMU KAEK 2025/638). Owing to the retrospective design of the study, the requirement for informed consent was waived by the Ethics Committee.The study was conducted in accordance with the ethical principles of the Declaration of Helsinki.

Eligible cases were identified through the hospital’s electronic medical record system and archived patient files. Patients with incomplete obstetric or radiological data, or those whose spinal deformity was secondary to acute trauma, malignancy, or infection, were excluded from the study. Demographic, clinical, obstetric, and neonatal data were retrieved from the hospital information management system and archival records. Recorded variables included demographic characteristics (maternal age, gravidity, parity, gestational age at delivery, and body mass index); spinal deformity–related characteristics (type and localization of the deformity, Cobb angle, and history of prior spinal surgery); obstetric variables [mode and method of delivery, fetal presentation, preeclampsia, postpartum hemorrhage, preterm birth, and intrapartum complications]; anesthetic management (type of anesthesia administered during delivery: spinal, epidural, or general); neonatal outcomes (gestational age at birth, birth weight, 1- and 5-minute Apgar scores, and need for neonatal intensive care unit admission); maternal systemic parameters (length of hospital stay, need for adult intensive care unit admission, and postpartum complications such as ileus or atelectasis); and results of systemic evaluation tests, including echocardiography and pulmonary function tests, when available. To ensure clinical clarity, major obstetric complications were strictly defined. Fetal growth restriction (FGR) was defined as an estimated fetal weight below the 10th percentile for gestational age. Postpartum hemorrhage (PPH) was defined clinically as an estimated blood loss exceeding 1000 mL following cesarean delivery or requiring blood transfusion, rather than merely reflecting routine surgical blood loss.

**Fig. 1:**
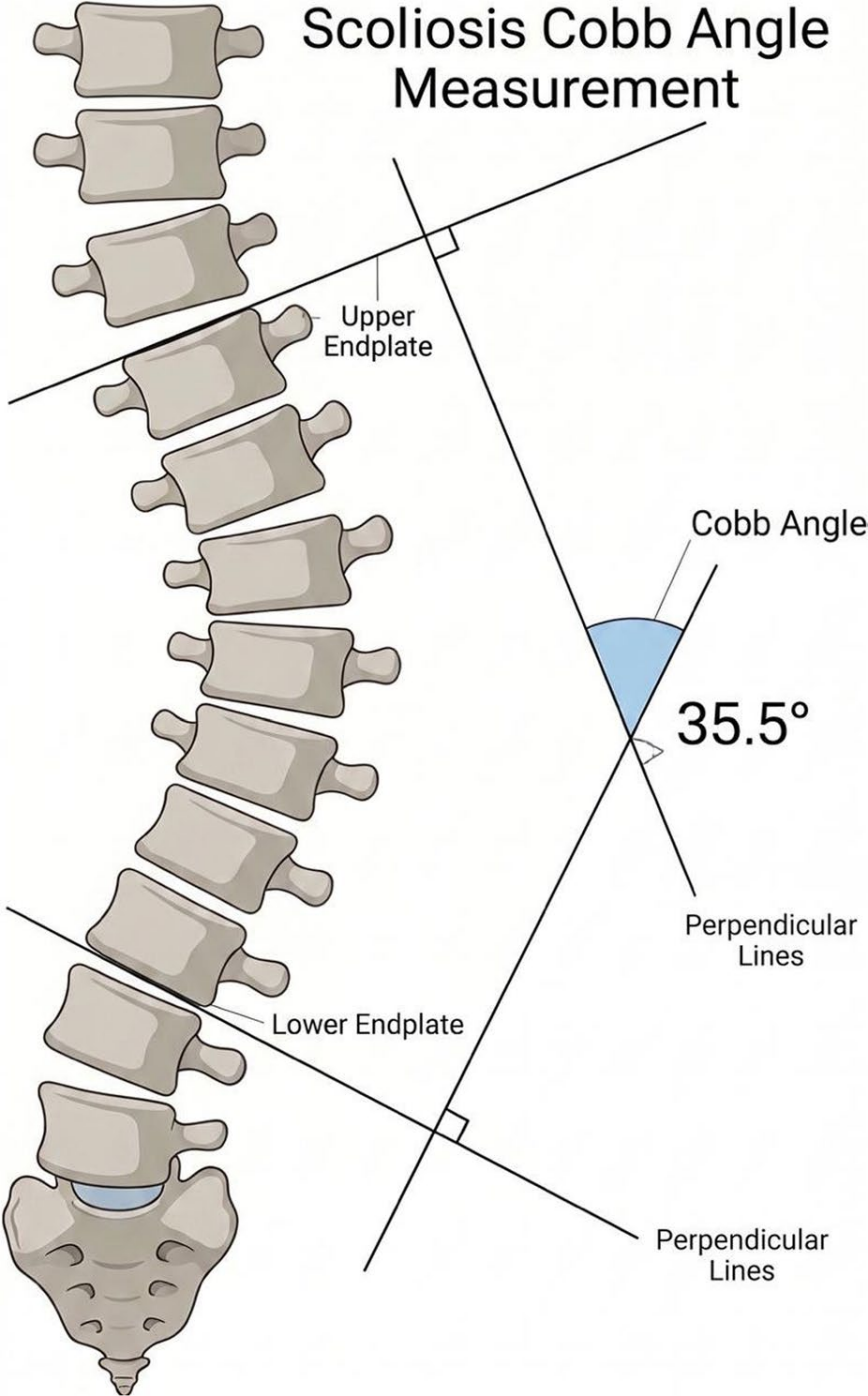
Measurement of Cobb angle on spinal radiograph

**Fig. 2:**
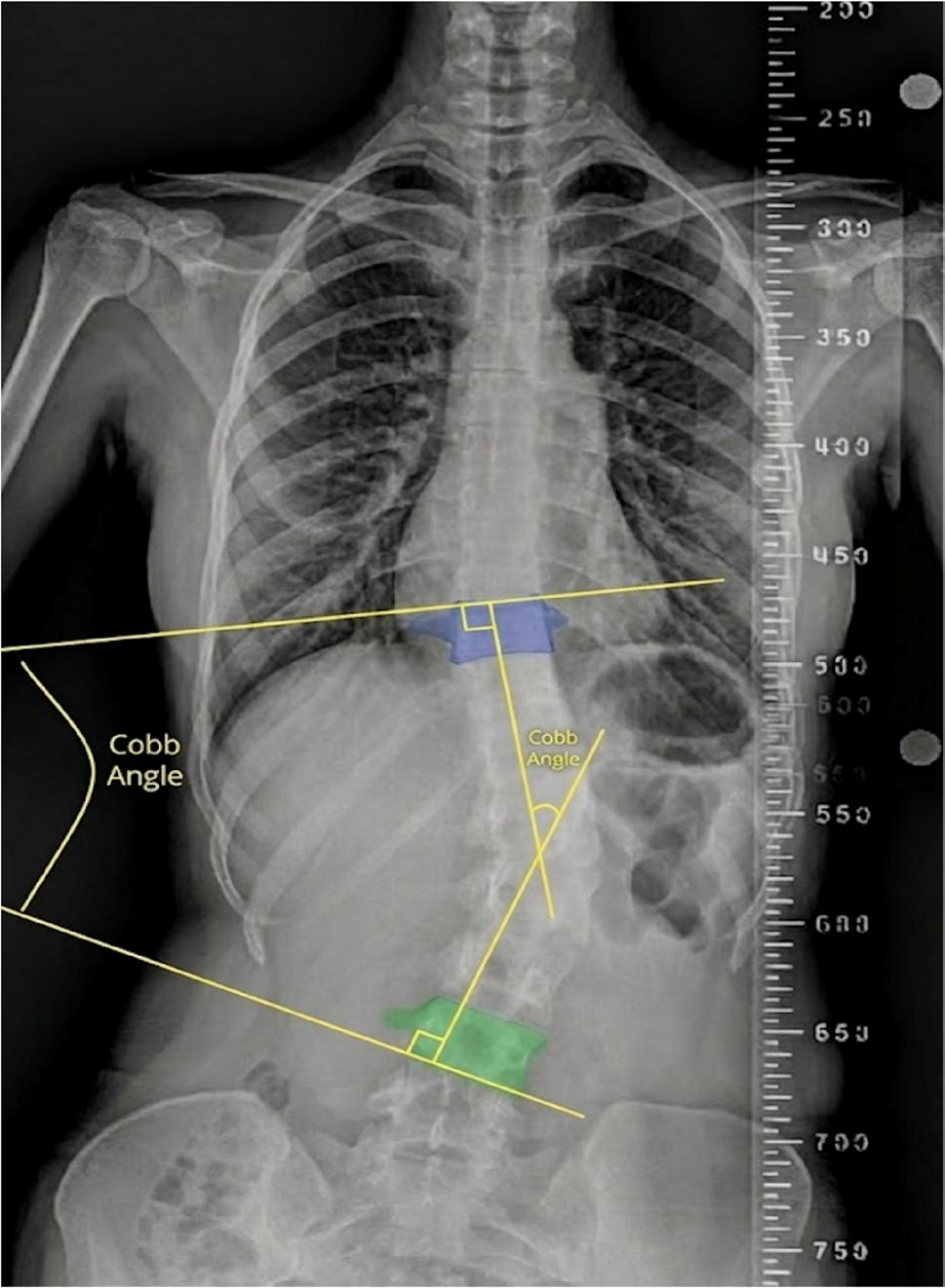
Measurement of scoliotic Cobb angle (coronal plane)

**Fig. 3:**
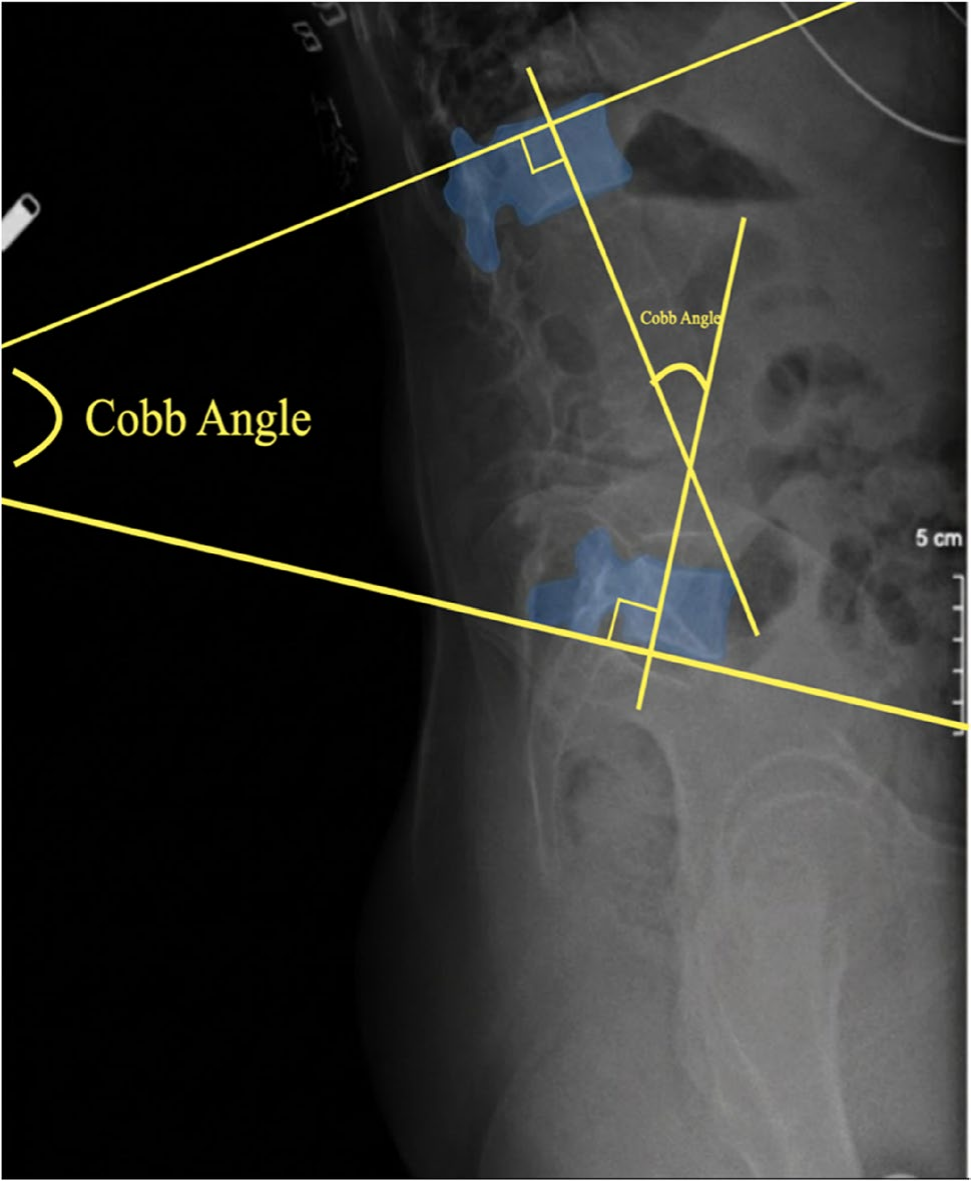
Measurement of kyphotic Cobb angle (sagittal plane)

The severity of spinal curvature was assessed using the Cobb angle. Measurements were performed on the most recent pre-pregnancy standing posteroanterior (PA) and lateral spinal radiographs available for each patient. Upon retrospective evaluation of the radiological chronological data, all utilized pre-pregnancy radiographs were confirmed to have been obtained within one year prior to conception (range: 2 to 12 months). Furthermore, for the 14 patients with a history of prior spinal surgery, the measurements were strictly performed on their most recent post-operative radiographs to accurately reflect their actual spinal alignment and functional status during the index pregnancy. The Cobb angle was calculated as the angle formed by the intersection of perpendicular lines drawn from the superior endplate of the uppermost (cephalad) vertebra and the inferior endplate of the lowermost (caudal) vertebra involved in the curvature (Fig. 1) [8]. In the clinical risk stratification and follow-up of pregnant patients with kyphoscoliosis, the primary parameter was the scoliosis Cobb angle measured on the PA radiograph. The calculation of the scoliotic Cobb angle from the PA view, reflecting coronal plane deformity, is illustrated in Fig. 2, while the calculation of the kyphotic Cobb angle from the lateral radiograph, representing sagittal plane deformity, is shown in Fig. 3. The kyphotic Cobb angle was utilized in cases with prominent sagittal deformity to aid in the assessment of maternal respiratory function and peripartum anesthetic management. For analytical purposes, patients were stratified into three severity subgroups based on their measured Cobb angles to evaluate the impact of deformity severity on clinical outcomes: mild (< 20°), moderate (20–40°), and severe (> 40°).

All statistical analyses were performed using IBM SPSS Statistics for Windows, Version 27.0 (IBM Corp., Armonk, NY, USA). Continuous variables were summarized as mean ± standard deviation (SD) for normally distributed data and as median (interquartile range, IQR) for non-normally distributed data. Categorical variables were expressed as counts (n) and percentages (%). The normality of continuous variables was assessed using both visual methods (histograms and probability plots) and analytical methods (skewness and kurtosis values, Kolmogorov–Smirnov test, and Shapiro–Wilk test). For comparisons between two groups, independent samples t-test was used for normally distributed variables, and the Mann–Whitney U test for non-normally distributed variables. For categorical variables, the chi-square test or Fisher’s exact test was applied, as appropriate. When comparisons involved more than two groups, one-way ANOVA was used for normally distributed variables, and the Kruskal–Wallis H test for non-normally distributed variables. A p-value of < 0.05 was considered statistically significant. Given the relatively small sample size and the descriptive nature of the study, all results are presented primarily using descriptive statistics, with inferential analyses included to support group comparisons where appropriate.

## Results

During the study period, a total of 38 pregnant women with a diagnosis of kyphoscoliosis were initially identified from the hospital records. Following a detailed review of their medical histories, 5 patients were excluded from the study because their spinal deformity was secondary to non-idiopathic causes, specifically infection (*n* = 3) and malignancy (*n* = 2). Consequently, a total of 33 women who met all inclusion criteria and possessed complete obstetric and radiological data were included in the final analysis.In the majority of cases, the diagnosis of kyphoscoliosis had been established during childhood, with a mean age at diagnosis of 9.90 ± 3.14 years. All pregnancies resulted from spontaneous conception; no assisted reproductive techniques were used, and no multiple gestations were observed. Spinal surgery had been performed prior to pregnancy in 42% of the cohort, with a mean age at surgery of 16.4 ± 3.2 years. Detailed demographic and clinical characteristics of the study population are presented in Table 1.

**Table 1:** Baseline demographic and clinical characteristics of pregnant women with kyphoscoliosis

Parameter	Results
Maternal age, years (Mean±SD)	30.67±5.24
Parity *n* (%)	
Nulliparous	22 (66%)
Parous	11 (33%)
Body mass index, (kg/m²)	22.83±2.48
Smoking status *n* (%)	2 (6%)
Age at diagnosis, years (Mean±SD)	9.90±3.14
History of spinal surgery, *n* (%)	
Yes	14 (42%)
No	19 (58%)
Age at spinal surgery, years (Mean±SD)	16.4 ± 3.2
Cardiac evaluation [echocardiography], *n* (%)	
Normal	12 (36%)
Abnormal	4 (12%)
Pulmonary function test, *n* (%)	
Normal	8 (24%)
Abnormal	2 (6%)
Mode of conception *n* (%)	
Spontaneous conception	33 (100%)
Assisted reproductive technology	0 (0%)

In all cases, the etiological classification was identified as adolescent idiopathic scoliosis (AIS), and a kyphoscoliotic pattern was present in all patients. Deformities predominantly involved the cervical spinal segments (*n* = 24, 72.7%), whereas thoracolumbar (*n* = 4, 12.1%) and cervicothoracic (*n* = 5, 15.2%) involvement was comparatively less frequent. The severity of curvature demonstrated a wide distribution, with Cobb angles ranging from 11° to 54°. The mean Cobb angle across the cohort was 24.94 ± 12.16°. Most patients exhibited mild (*n* = 14, 42.4%) or moderate (*n* = 14, 42.4%) curvature, whereas severe deformity (> 40°) was observed in a limited number of cases (*n* = 5, 15.2%).Detailed case-based information regarding deformity characteristics and curvature severity is provided in Table 2.

**Table 2:** Obstetric, maternal, and neonatal outcomes in pregnancies with kyphoscoliosis

Case	Etiology	Deformity Type	Curve Localization	Cobb Angle (°)	Mode of Delivery	Gestational Age at Delivery (weeks)	Birth Weight (g)	Anesthesia Type	Obstetric Complications	Maternal Complications	Length of Hospital Stay (Days)	ICU Admission	NICU Admission	Neonatal Complications
1	AIS	KS	Cervical	53	C/S	34	1875	GA	FGR	Hypoxemia / oxygen requirement	4	Yes	Yes	TTN
2	AIS	KS	Cervical	16	C/S	38	3070	GA	Malpresentation (breech)	None	3	No	No	None
3	AIS	KS	Cervical	26	C/S	36	3030	GA	Malpresentation (transverse)	None	4	No	No	None
4	AIS	KS	Cervical	35	C/S	38	3475	GA	None	None	3	No	No	None
5	AIS	KS	Cervical	46	C/S	38	2570	GA	None	Difficult airway management	3	No	No	None
6	AIS	KS	Cervical	16	C/S	36	2820	GA	None	None	3	No	No	None
7	AIS	KS	Cervical	12	C/S	37	3335	GA	Oligohydramnios	None	3	No	No	None
8	AIS	KS	Cervical-Thoracic	15	C/S	28	1790	GA	None	PPH Ileus	12	No	Yes	RDS
9	AIS	KS	Cervical	26	C/S	33	2450	GA	None	Difficult or failed neuraxial anesthesia	4	No	Yes	None
10	AIS	KS	Cervical	30	C/S	36	2270	GA	Malpresentation (breech)	None	4	No	Yes	TTN
11	AIS	KS	Cervical	16	C/S	32	3830	GA	None	PPH	3	No	Yes	RDS
12	AIS	KS	Cervical-Thoracic	54	C/S	39	3000	GA	None	Hypoxemia / oxygen requirement	3	Yes	No	None
13	AIS	KS	Cervical	15	C/S	37	3620	GA	None	None	3	No	No	None
14	AIS	KS	Cervical	30	C/S	38	2790	GA	Polyhydramnios	None	3	No	No	None
15	AIS	KS	Thoracolumbar	20	C/S	35	2940	GA	None	PPH	8	Yes	Yes	None
16	AIS	KS	Cervical	24	C/S	36	2550	GA	FGR	None	3	No	Yes	None
17	AIS	KS	Cervical	18	C/S	34	2580	GA	None	None	3	No	Yes	None
18	AIS	KS	Cervical	25	C/S	37	2590	GA	Oligohydramnios, Malpresentation [transverse]	None	2	No	No	None
19	AIS	KS	Cervical	25	C/S	37	2790	GA	None	None	3	No	No	None
20	AIS	KS	Thoracolumbar	12	C/S	38	2345	GA	Polyhydramnios	Ileus	3	No	Yes	TTN
21	AIS	KS	Cervical	48	VB	38	3750	-	Preeclampsia GDM	Difficult airway management	3	No	No	None
22	AIS	KS	Cervical	15	C/S	39	2185	GA	GDM Polyhydramnios Malpresentation [breech]	None	3	No	Yes	Congenital anomalies; Spina bifida
23	AIS	KS	Cervical	14	C/S	36	2100	GA	Placenta previa	PPH	3	No	Yes	None
24	AIS	KS	Cervical	20	C/S	33	1780	GA	Preeclampsia	PPH	4	No	Yes	Cerebral palsy; meningitis-related neonatal death
25	AIS	KS	Cervical-Thoracic	15	C/S	34	2875	GA	None	None	3	No	Yes	None
26	AIS	KS	Cervical	42	C/S	37	1995	GA	Malpresentation [breech]	Difficult or failed neuraxial anesthesia	3	No	Yes	Congenital diaphragmatic hernia; neonatal death
27	AIS	KS	Cervical	36	C/S	34	1430	GA	None	Difficult or failed neuraxial anesthesia	3	No	Yes	Neonatal sepsis-related death
28	AIS	KS	Cervical-Thoracic	15	C/S	35	3010	GA	None	None	3	No	Yes	None
29	AIS	KS	Cervical-Thoracic	18	VB	38	2920	-	Polyhydramnios	PPH	3	No	No	None
30	AIS	KS	Thoracolumbar	21	C/S	37	2985	S	None	None	3	No	No	None
31	AIS	KS	Cervical	28	C/S	37	1810	GA	None	None	2	No	Yes	None
32	AIS	KS	Cervical	11	C/S	35	2915	GA	None	PPH	2	No	Yes	None
33	AIS	KS	Thoracolumbar	26	C/S	38	2810	S	None	None	2	No	No	None

*AIS* Adolescent idiopathic scoliosis, *B* Breech Presantation, *C* Cephalic Presentation, *CS* Cesarean Section, *GA* General Anesthesia, *GDM* Gestational Diabetes, *FGR* Fetal growth restriction, *I* Ileus, *KS* Kyphoscoliosis, *P* Preeclampsia, *PPH* Postpartum Hemorrhage, *RDS* Respiratory distress syndrome, *S* Spinal Anesthesia,  *T* Transverse Lie, *TTN* Transient Tachypnea of the Newborn, *VB* Vaginal Birth

With respect to mode of delivery, cesarean section was performed in 31 of 33 cases (94%), while vaginal delivery occurred in only two cases (6%). Deliveries took place between 28 and 39 weeks of gestation, with a mean gestational age at delivery of 36.0 ± 2.3 weeks; a substantial proportion of pregnancies resulted in late preterm or term birth Regarding anesthetic management, general anesthesia was administered in the majority of cases; spinal anesthesia was preferred in only two patients.Among obstetric complications, 1 woman developed preeclampsia, and gestational diabetes occurred in two women. Also, 2 fetuses had fetal growth restriction, and postpartum hemorrhage was documented in seven cases.

Overall, maternal complications were limited, with most patients having a hospital length of stay ranging from 2 to 4 days. Ileus developed in two cases, resulting in prolonged hospitalization. The most frequent maternal complication was postpartum hemorrhage, which occurred in seven patients. Hypoxemia with oxygen requirement and difficult airway management were each observed in two cases, while difficult or failed neuraxial anesthesia was documented in three patients. Admission to the adult intensive care unit was required for three patients. Neonatal assessment revealed that the mean birth weight of the newborns was 2675.3 ± 593.45 g. Neonatal assessment revealed that 24 of 33 newborns (72.7%) had no complications, whereas at least one neonatal complication was identified in nine newborns (27.3%). The most common neonatal morbidities were respiratory in nature, with transient tachypnea of the newborn observed in three cases and respiratory distress syndrome in two cases. Neonatal mortality occurred in three cases (9.1%), attributable to neonatal sepsis, meningitis-related complications, and congenital diaphragmatic hernia.

To comprehensively evaluate the clinical heterogeneity of our cohort, subgroup analyses were performed based on the history of prior spinal surgery (Table 3) and the severity of the Cobb angle (Table 4). When comparing patients with (*n* = 14) and without (*n* = 19) prior spinal surgery, the incidence of obstetric complications was notably higher in the prior surgery group (64.3% vs. 31.6%), showing a strong trend toward statistical significance (*p* = 0.062). However, anesthetic management, mode of delivery, and neonatal outcomes did not differ significantly between these two groups.

**Table 3:** Comparison of obstetric and neonatal outcomes according to history of previous spinal surgery

Parameter	Prior spinal surgery (*n* = 14)	No prior surgery (*n* = 19)	*P* value
Cesarean delivery	12 (85.7%)	19 (100%)	0.172^2^
Vaginal birth	2 (14.3%)	0 (0%)	
General anesthesia	11 (78.6%)	18 (94.7%)	0.233^1^
Neuraxial anesthesia	1 (7.1%)	1 (5.3%)	
ICU admission	1 (7.1%)	2 (10.5%)	1^2^
NICU admission	7 (50%)	11 (57.9%)	0.653^1^
Neonatal complications	4 (28.6%)	5 (26.3%)	1^2^
Obstetric complication	9 (64.3%)	6 (31.6%)	0.062^1^
Maternal complication	5 (35.7%)	10 (52.6%)	0.335^1^
Preterm delivery (< 37w)	6 (42.9%)	10 (52.6%)	0.579^1^
Birth Weight (Mean ± SD)	2686.7 ± 600.4	2666.8 ± 604.5	0.926^3^
Length of hospitalization (Median - IQR)	3 (3–3)	3 (3–4)	0.843^4^

1 Chi-square test, 2 Fisher’s exact test, 3 Independent samples t-test,4 Mann-Whitney U

**Table 4:** Outcomes according to Cobb angle severity

Parameter	Mild (< 20°) (*n* = 14)	Moderate (20–40°) (*n* = 14)	Severe (> 40°) (*n* = 5)	*P* value
Cesarean delivery	13 (92.9%)	14 (100%)	4 (80%)	0.267^1^
Vaginal birth	1 (7.1%)	0 (0%)	1 (20%)	
General anesthesia	13 (92.9%)	12 (85.7%)	4 (80%)	0.260^1^
Neuraxial anesthesia	0 (0%)	2 (14.3%)	0 (0%)	
ICU admission	0 (0%)	1 (7.1%)	2 (40%)	**0.027** ^**1**^
NICU admission	9 (64.3%)	7 (50%)	2 (40%)	0.583^1^
Neonatal complications	4 (28.6%)	3 (21.4%)	2 (40%)	0.718^1^
Obstetric complication	6 (42.9%)	6 (42.9%)	3 (60%)	0.778^1^
Maternal complication	6 (42.9%)	4 (28.6%)	5 (100%)	**0.022** ^1^
Preterm delivery (< 37w)	6 (42.9%)	7 (50%)	4 (80%)	0.354^1^
Birth Weight (Mean ± SD)	2813.9 ± 575.8	2550 ± 561.2	2638 ± 769.3	0.509^3^
Length of hospitalization (Median-IQR)	3 (3–3)	3 (2,75 − 4)	3 (3–4)	0.768^4^

1 Chi-square test, 2 Fisher’s exact test, 3 Independent samples t-test, 4 Mann-Whitney U

Bold values indicate statistically significant results (*p*< 0.05)

Furthermore, stratification by deformity severity into mild (< 20°, *n* = 14), moderate (20–40°, *n* = 14), and severe (> 40°, *n* = 5) groups revealed highly significant clinical correlations. The rate of maternal complications was significantly higher in the severe group (100%) compared to the mild (42.9%) and moderate (28.6%) groups (*p* = 0.022). Consequently, maternal intensive care unit (ICU) admissions were also significantly concentrated in patients with severe Cobb angles (40%) compared to those with moderate (7.1%) and mild (0%) deformities (*p* = 0.027). Neonatal outcomes and birth weights did not demonstrate statistically significant differences across severity groups.

The indications for cesarean delivery were primarily driven by obstetric necessities rather than institutional preference or anesthetic limitations. The most frequent explicit obstetric indications for cesarean delivery included fetal malpresentation (*n* = 6, 19.3% of cesarean cases), fetal growth restriction (*n* = 2, 6.4%), and placenta previa (*n* = 1, 3.2%); whereas the remaining majority of cesarean sections were performed due to suspected cephalopelvic disproportion secondary to kyphoscoliosis-induced pelvic skeletal distortion.

## Discussion

In this study, the clinical course of 33 pregnant women diagnosed with kyphoscoliosis was evaluated. Pregnancy in the presence of kyphoscoliosis represents a multidisciplinary clinical condition with not only obstetric but also respiratory, cardiovascular, and anesthetic implications. When the three-dimensional spinal deformity is combined with the increased physiological demands of pregnancy, maternal adaptive capacity may be compromised, rendering peripartum management more complex. Therefore, pregnancies complicated by kyphoscoliosis should be regarded as a distinct clinical entity requiring individualized surveillance and delivery strategies beyond standard obstetric care. In our cohort, despite relatively low mean Cobb angles, the observed patterns in mode of delivery and timing of birth suggest that assessing pregnancy risk solely on the basis of curvature severity is insufficient. Structural characteristics such as the direction and rotational component of the deformity, as well as the functional capacity of the thoracic cage, appear to be at least as influential as the Cobb angle in guiding obstetric decision-making. These findings are consistent with data reported in contemporary series by Chopra et al., which emphasize that maternal and perinatal outcomes in kyphoscoliosis are influenced by multidimensional factors [9]. This evidence, as well as other studies in the literature, supports the need for a holistic and individualized approach in the management of this patient population [10]. 

Within this clinical framework, anesthetic management represents one of the most critical components of obstetric decision-making in pregnancies complicated by kyphoscoliosis. In our series, the predominant use of general anesthesia cannot be attributed solely to clinician preference, but rather suggests that the anatomical distortion associated with kyphoscoliosis directly limits the feasibility of neuraxial anesthesia. Displacement of intervertebral spaces, loss of midline landmarks, and asymmetric dural positioning due to rotational deformity render epidural or spinal access technically challenging. Previous studies have demonstrated that increasing Cobb angles are associated with higher rates of neuraxial anesthesia failure and subsequent conversion to general anesthesia [11, 12]. Although Chan et al. did not obtain results similar to ours, they also examined thoracic and lumbar angles and associated clinical outcomes, as we did in our study [13]. In addition, the substantial proportion of patients in our cohort with a history of spinal surgery may have further restricted neuraxial anesthesia feasibility, particularly due to instrumentation and postoperative fibrosis. Collectively, these observations support the need for individualized anesthetic planning in pregnant women with kyphoscoliosis, with careful consideration of anatomical and neurological risks. Our statistical subgroup analyses yielded clinically relevant findings, particularly regarding deformity severity. Patients with severe Cobb angles (> 40°) had significantly higher rates of overall maternal complications and required postoperative ICU admission more frequently compared to those with milder deformities. These findings support the hypothesis that advanced spinal curvature may compromise maternal cardiopulmonary reserve, potentially reducing physiological tolerance to the hemodynamic changes associated with pregnancy and delivery. Interestingly, although the vast majority of patients with prior spinal surgery required general anesthesia, the overall statistical comparison of anesthetic choices and neonatal outcomes between the surgery and non-surgery groups did not reveal significant differences. Furthermore, while a notably higher rate of obstetric complications was observed in the prior surgery group, this difference demonstrated a strong trend rather than definitive statistical significance. This suggests that baseline anatomical alterations inherently contribute to obstetric risk, regardless of previous surgical correction.

Maternal complications were clinically significant, with postpartum hemorrhage representing the most frequently encountered adverse outcome. In the context of kyphoscoliosis, pelvic muscle asymmetry, hemodynamic alterations secondary to thoracic deformity, and technical challenges during surgical delivery may collectively contribute to an increased risk of hemorrhage. There is a paucity of data specifically evaluating postpartum hemorrhage in pregnancies complicated by kyphoscoliosis. However, several factors known to increase the risk of postpartum hemorrhage, including prolonged operative time, technically challenging surgery, and underlying cardiopulmonary compromise, may be more frequently encountered in this patient population. Therefore, an increased risk of postpartum hemorrhage may be anticipated, particularly in patients with more severe deformities.In the postoperative period, gastrointestinal complications were infrequent; however, ileus occurred in two cases, resulting in prolonged hospital stay. From a neonatal perspective, the relatively high rate of neonatal intensive care unit admission indicates the persistence of fetal risk in this population. Respiratory morbidity related to prematurity, fetal growth restriction, and congenital anomalies have been identified as the principal neonatal complications in the literature [14]. Consistent with these reports, a remarkable finding in our cohort is the high incidence of preterm deliveries. Unlike spontaneous preterm labor typical in the general obstetric population, the high rate in kyphoscoliosis patients is frequently iatrogenic or driven by mechanical constraints. Severe spinal deformity may contribute to reduced abdominal space, potentially limiting uterine expansion. This mechanical constraint, together with progressive maternal respiratory compromise in late pregnancy, may partially explain the increased rate of medically indicated preterm deliveries in this population. Therefore, the decision for preterm delivery in these patients represents a delicate balance between optimizing fetal maturation and preventing maternal respiratory failure. Notably, the neonatal mortalities observed in our cohort were attributable to independent neonatal pathologies rather than a direct consequence of maternal spinal deformity.

Given these multifaceted maternal and neonatal risks, our findings support the importance of comprehensive preconception counseling for women with kyphoscoliosis. A multidisciplinary team comprising maternal-fetal medicine specialists, anesthesiologists, and pulmonologists should evaluate the baseline cardiopulmonary reserve and functional status of these patients before conception. During pregnancy, tailored delivery planning should be formulated early in the third trimester, explicitly detailing the anesthetic strategy, anticipating potential difficult airway scenarios, and establishing a clear threshold for surgical intervention in the event of maternal respiratory deterioration.

The principal strength of this study lies in its comprehensive evaluation of obstetric, anesthetic, and neonatal outcomes, bolstered by targeted statistical subgroup analyses based on deformity severity and surgical history. This dual approach highlights the multidimensional nature of clinical management in this population. The comparability of our findings with classical and contemporary series in the literature further enhances the clinical relevance of the results. Nevertheless, the retrospective, single-center design and the limited overall sample size restrict the broad generalizability of the findings. While our subgroup analyses successfully revealed critical clinical nuances and statistically significant trends, the relatively small number of patients within specific subsets (such as the severe deformity group, *n* = 5) may limit the statistical power to detect smaller differences. Therefore, these subgroup findings should be interpreted as strong preliminary signals that warrant validation in future large-scale cohorts, rather than definitive universal conclusions. Furthermore, the absence of a control or reference group comprising the general obstetric population remains a notable limitation; without such a comparator, it is challenging to definitively ascertain the extent to which the reported outcomes in our cohort differ meaningfully from baseline population risks. Additionally, our study focused exclusively on peripartum and immediate postpartum outcomes.

## Conclusions

Pregnancies complicated by kyphoscoliosis represent a distinct clinical entity that may be associated with an increased potential for obstetric, anesthetic, and neonatal complications. This highlights the imperative for highly patient-specific care, regardless of deformity severity. Based on our observations, we speculate that pregnancy and delivery management might benefit from incorporating multidimensional clinical parameters—potentially including thoracic functional capacity, history of spinal surgery, and maternal physiological reserve—rather than relying solely on radiological measurements.Ultimately, early multidisciplinary evaluation and proactive perinatal care in appropriately equipped tertiary centers are the cornerstones of optimizing outcomes for this vulnerable population. Future large-scale, prospective, multicenter studies are warranted to further refine evidence-based management strategies.

## Data Availability

The datasets generated and/or analyzed during the current study are not publicly available due to patient confidentiality but are available from the corresponding author on reasonable request.

## Abbreviations

AIS: Adolescent idiopathic scoliosis
BMI: Body mass index
CS: Cesarean section
GA: General anesthesia
GDM: Gestational diabetes
FGR: Fetal growth restriction
ICU: Intensive care unit
KS: Kyphoscoliosis
NICU: Neonatal intensive care unit
PPH: Postpartum hemorrhage
RDS: Respiratory distress syndrome
S: Spinal Anesthesia
TTN: Transient tachypnea of the newborn
VB: Vaginal birth
